# Sport and mental health performance optimization in an adolescent gymnast: A case evaluation

**DOI:** 10.3389/fspor.2023.1018861

**Published:** 2023-02-06

**Authors:** Davy Phrathep, Brad Donohue, Brenna N. Renn, John Mercer, Daniel N. Allen

**Affiliations:** ^1^ Department of Psychology, University of Nevada, Las Vegas, NV, United States; ^2^ Department of Kinesiology and Nutrition Sciences, University of Nevada, Las Vegas, Nevada, United States

**Keywords:** athlete mental health, sport performance, adolescent athletes, intervention - behavioral, latina adolescent

## Abstract

The Optimum Performance Program in Sports (TOPPS) is a multi-component, sport-specific Family Behavior Therapy that has demonstrated improved sport performance, relationships, and mental health outcomes in adult and adolescent athletes with, and without, diagnosed mental health disorders in clinical trials. The current case trial demonstrates successful implementation of a novel component of TOPPS (i.e., talk aloud optimal sport performance imagery leading to dream mapping) in a biracial Latina and White adolescent gymnast without a mental health diagnosis. The participant demonstrated significant improvements from baseline to both post-treatment and 3-month follow-up in severity of mental health functioning, factors interfering with sports performance, and her relationships with teammates, coaches, and family. Results suggest it may be possible to optimize mental health through sport performance optimization.

## 1. Introduction

The Optimum Performance Program in Sports (TOPPS) is a family-supported, sport-specific cognitive-behavioral intervention that was adapted from Family Behavior Therapy (1). As extensively reviewed in a published treatment manual (2), TOPPS was developed with support from the National Institutes of Health and focuses on the concurrent optimization of mental health and sport performance through the implementation of cognitive-behavioral intervention components (e.g., motivational enhancement, functional analysis assessment, thought management, self- and environmental control, appreciation exchange, positive and negative assertion skills training). TOPPS is typically implemented in 12 to 16 outpatient meetings each lasting 60 min by mental health and sport performance professionals who are explicitly trained in TOPPS. Athletes usually pursue TOPPS to optimize their performance in sports or life outside of sports. Intervention components are prioritized from a menu of options and used to facilitate thoughts and actions that are consistent with optimal performance in relationships, creativity, sport/physical health, mental strength and stability, and work/school.

Clinical trials evaluating TOPPS have involved collegiate (3–8) and adolescent (9–12) athletes participating in various sports (e.g., American football, soccer/football, track and field, hockey, softball/baseball, cheer, swimming, boxing, tennis) and formally assessed for mental health disorders. The results of these studies consistently indicate significant improvements in mental health and relationships with coaches, family members and teammates, and factors that interfere with sport performance. In the Donohue et al. (13) controlled clinical trial, as baseline mental health severity increased, outcome improvements with participants receiving TOPPS, as compared with traditional campus counseling, became more pronounced; and outcome improvements for participants receiving TOPPS was positively correlated with the number of athletes' supportive others involved in programming.

The current case trial examined the impact of a novel component of TOPPS (i.e., talk aloud performance optimization imagery combined with dream mapping or “Performance Mapping” (see Appendix B) in a biracial Latina and White adolescent gymnast. This athlete was referred to TOPPS by her father to ultimately optimize her performance in gymnastics. The athlete agreed to focus her programming on a novel application of Dream Mapping, called “Performance Mapping.” Dream Mapping has been successfully used to treat nightmares (14); it involves imagination of a scripted pleasant dream prior to bedtime. In Performance Mapping the athlete is taught to imagine sequentially experienced thoughts and actions that are consistent with optimum performance in a performance scenario; and to then sleep. Impactful moments of time before (e.g., warm-up), during (e.g., tennis serve), and/or after (e.g., shower) the scenario, as well as factors that optimize performance (e.g., thoughts, routines, nutrition) are derived using the Performance Timeline Worksheet (see Appendix A). For the respective time period(s), athletes are taught to sequentially describe in the present tense, and in first person, so the provider can provide directive feedback in real-time, their performance of optimum thoughts and actions. To expand on traditional mindfulness approaches that focus on describing sensations during visualization (15), athletes are taught to perceive their senses as contributing to optimal performance during these trials (e.g., “I smell the chalk in the gym and it lets me know it’s time to dominate”). After these performance scripts are developed and practiced in meetings, the athlete is assigned to imagine them immediately prior to bed.

### 1.1. Study design

The proposed study utilized an A/B case study design with 3-month follow-up. It was hypothesized that after receiving the talk aloud performance optimization imagery (i.e., Performance Mapping), the participant would demonstrate significant improvements in mental health, relationships, and common interferences with sport performance according to her responses to standardized measures completed at baseline, immediately post-intervention and at three-month follow-up.

## 2. Patient information

Nina is a 13-year-old biracial Latina and White female middle school competitive club gymnast. She was referred to TOPPS by her father to improve her gymnastics performance, which he believed was being negatively impacted because she was “dwelling” on her mistakes and lacked confidence in sports.

### 2.1. Presenting complaints

During intake, Nina reported her lack of confidence made her overly self-conscious when others watched her perform. She expressed a desire to improve her self-confidence, motivation, and positive mindset.

### 2.2. History

Nina's father indicated that her negative self-talk adversely affected her gymnastic performances. Nina mentioned that she was fearful of repeating mistakes in future performances, experienced heightened self-consciousness in competitions, and became easily frustrated when learning gymnastics new skills.

## 3. Diagnostic assessment

After referral and consent, Nina was administered a battery of standardized tests and measures by a trained technician one week prior to intervention, immediately following intervention completion, and three months after intervention completion. All measures have been psychometrically validated and have been used in adolescent athletes:

### 3.1. Diagnostic interview

#### 3.1.1. Kiddie – schedule for affective disorders and schizophrenia for school children aged 6–18 years old DSM-5

This semi-structured interview was used to assess psychiatric symptoms consistent with the Diagnostic and Statistical Manual of Mental Disorders (5th ed.) (16). Inter-rater agreement of the K-SADS with similar measures is high (range: 93% to 100%), and it has high test-retest reliability and concurrent validity in youth (17).

### 3.2. Primary measures

#### 3.2.1. The symptoms check-list-90-revised (SCL-90-R)

This 90-item measure is a widely utilized scale for general psychiatric symptoms, has been normed on adolescent populations and has demonstrated acceptable internal consistency and test-retest reliability (18, 19).

#### 3.2.2. Sports interference checklist (SIC)

This 40-item measure includes three inventories used to assess factors known to interfere with sports training, sports competition, and life outside of sports (20). The SIC has demonstrated excellent factor structure, internal consistency and convergent validity (20), and has predicted psychiatric symptom severity (21) in athletes.

### 3.3. Secondary measures

#### 3.3.1. Student athlete relationship instrument (SARI)

This 63-item measure assesses sport-specific problems in relationships with families, coaches, teammates, and peers. The SARI has demonstrated high internal consistency and criterion-related validity (22), and reliably predicts mental health symptom severity in athletes (23).

#### 3.3.2. Overall happiness with family, coaches, teammates, and peers

This 4-item measure utilizes a 0 to 100 scale of happiness (0 = completely unhappy, 100 = completely happy) (22). Items assess overall happiness in four relationships, e.g., coaches, teammates, family, and peers; these scales have demonstrated acceptable criterion-related validity in athletes (23).

#### 3.3.3. Client satisfaction questionnaire-8 (CSQ-8)

This 8-item (4-point scale) self-report questionnaire evaluates the quality of services received and has demonstrated high internal consistency and concurrent validity (24, 25).

### 3.4. Pre-intervention assessment results

Nina's results on the KSADS indicated that she did not evidence any formal mental health disorder, and her Depression, Paranoid Ideation, and Somatization subscales of the SCL-90-R were moderately elevated (see Table 1). She demonstrated Moderate score elevations on the SIC's Thoughts and Stress in Competition, and Injury in Training subscales (see Table 1).

**Table 1:** Pre-, post- and follow-up assessments of mental health and factors interfering with sport performance.

Scale	Pre-Intervention	Post-Intervention	Follow-up	Post-Intervention RCI	Follow-up Intervention RCI
**SCL-90-R**					
Psychoticism	44	33	33		
Obsessive-Compulsive	49	37	37		
Paranoid Ideation	57	31	31	2.11*	2.11*
Interpersonal Sensitivity	47	28	28	1.98*	1.98*
Anxiety	44	32	32		
Phobic Anxiety	44	39	39		
Depression	52	29	29	2.98*	2.98*
Hostility	49	33	33		
Somatization	58	28	30	2.86*	2.67*
Global Severity Index	50	22	23	2.89*	2.83*
**SIC Training**					
Total	54	43	39		
Thoughts and Stress	1.28	1.17	1.00		
Academic	1.00	1.00	1.00		
Injury	2.33	1.33	1.00	1.99*	2.01*
Team Relationships	1.00	1.00	1.00		
**SIC Competition**					
Total	51	43	39		
Thoughts and Stress	2.25	1.38	1.00	1.98*	2.02*
Academic and Adjustment	1.00	1.00	1.00		
Motivation	1.00	1.00	1.00		
Overly Confident/Critical	1.00	1.00	1.00		
Injury	1.00	1.00	1.00		
Pain	1.50	1.00	1.00		
**SIC Outside of Sport**					
Total	52	40	39		
**Yes Responses to Seeing a Professional**	-Overly concerned or worry too much about what others think about my performance	-N/A	-N/A		
**Student Athlete Relationship Instrument**					
Teammates Total Score	27	18	18		
Family Total Score	18	16	16		
Coaches Total Score	20	19	19		
Peers Total Score	15	10	10		
**Overall Happiness with Family, Coaches, Teammates and Peers**					
Family	100%	100%	100%		
Coaches	100%	100%	100%		
Teammates	90%	100%	100%		
Peers	90%	100%	100%		

SCL-90-R, symptom check-list-90-revised; SIC, sports interference checklist.

* Reliable change index (RCI) > 1.96 is considered significant. Significant RCIs are signified with an asterisk.

### 3.5. Case conceptualization

Nina did not meet DSM-5 criteria for a mental health disorder (26) in baseline. However, she endorsed sub-clinical depressive symptoms associated with a loss of motivation, decreased concentration, and low self-esteem. She reported anxiety symptoms related to fear of injury and judgment related to her sports performance. She reported her thoughts of being judged often led to maladaptive thinking patterns (e.g., “I need,” “I have to,” “I must”) that distracted her during performance situations. She reported feelings of embarrassment after poor performance across various domains of her life, and often “gave up” in practice. Her father sometimes negatively reinforced these behaviors by showing empathy for her concerns rather than focusing on their prevention.

## 4. Therapeutic intervention

**Figure 1:**
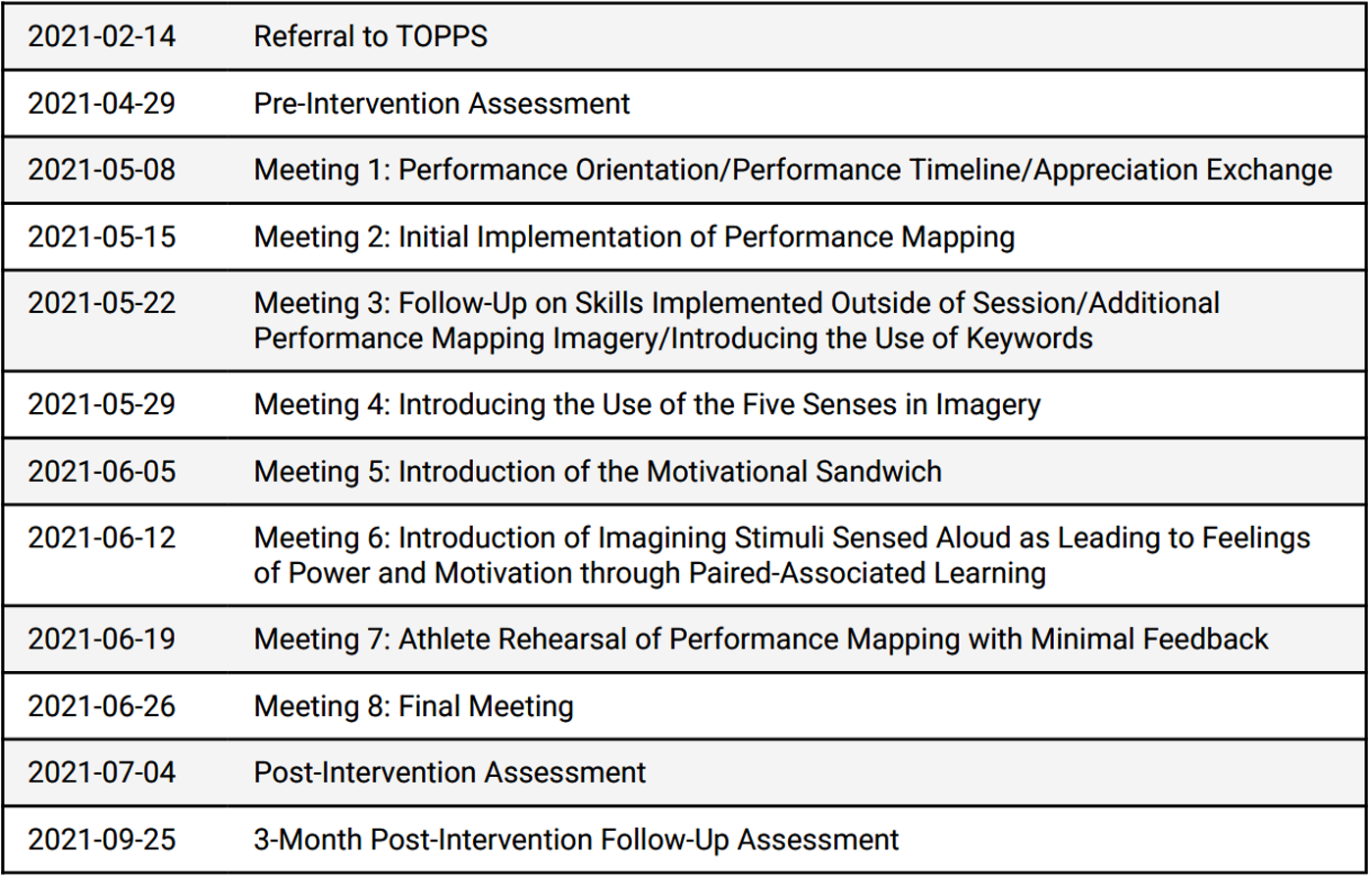
Treatment timeline.

The intervention involved eight performance meetings focused on optimizing her performance in sport scenarios (see Figure 1) using Performance Mapping; each meeting lasted 60 to 90 min. The provider was a clinical psychology doctoral student trained to perform TOPPS in a 3-day workshop, and to facilitate intervention integrity a licensed clinical psychologist provided one hour of office-based supervision each week of the study in addition to modeling novel skills during three of the performance meetings. This training is consistent with providers in previous clinical trial evaluations of TOPPS (see clinical trials mentioned above).

### 4.1. Meeting 1 (Nina, father, and mother)

During Meeting 1, a standardized orientation was conducted to provide a program overview, discuss the family's expectations for intervention, explain performance optimization theory, and gather information about overarching goals for programming. The Performance Timeline Worksheet (see Appendix A) was administered to teach Nina a standardized method of assessing performance situations. She indicated that her most recent performance situation was her floor routine, and the time period that most likely impacted this experience was during the routine. When asked to review this worksheet, she reported her “thoughts” most likely influenced her performance during that time period. For this time period, she was taught to sequentially imagine her floor routine, and during this imagination she was instructed to sequentially report, out-loud, what she believed were optimal actions and thoughts throughout the routine. During these reports, the provider immediately reinforced objective thinking patterns and positive nomenclature and instructed her to immediately “optimize” non-optimal thoughts. For instance, one time she reported, “I am not worried about what others think about me” and the provider instructed her to alternatively state, “I am focused on the task at hand, and I love my sport.”

Given Nina's expressed concern about being preoccupied with how others negatively judge her, an appreciation exchange was implemented at the end of Meeting 1. Nina's parents commented they appreciated her determination and caring personality towards her sport, friends, and family. Nina and her father expressed their appreciation for her mother's efforts in supporting the family, and Nina and her mother expressed appreciation for her father's consistent encouragement and commitment to the family. The provider assigned Nina to provide appreciations to others (e.g., coaches, friends, teammates, family) outside of meetings.

### 4.2. Meeting 2 (Nina and father)

The provider reviewed benefits of some of Nina's assigned appreciations with her teammates, family and coaches since last contact. She utilized the Performance Timeline Handout to identify impactful performance moments days, minutes and seconds leading up to bars training (e.g., the night before, breakfast, car ride). For instance, Nina chose seconds before stepping up to bars as an impactful moment and indicated that her thoughts, body/physical, and emotions were the most influential factors during this time. She was assisted in brainstorming optimal thoughts and behaviors for these moments, and to sequentially rehearse out loud in the first-person and present tense the generated optimal thoughts and behaviors (i.e., My body feels energized, and I'm feeling confident. I look up and…). Whenever non-optimal thoughts and actions were expressed (e.g., “I need to practice my breathing…”), the provider instructed her to restart her rehearsal and immediately optimize these statements; prompting correct responding whenever necessary (e.g., “I’m grateful my coaches are helping me learn diaphragmatic breathing.”). The provider also provided descriptive praise for optimal statements.

### 4.3. Meeting 3 (Nina and father)

Using the Performance Timeline Nina identified her standing back tuck as her optimal performance scenario. She identified her energy, motivation, emotions, and thoughts as the most influential factors, and she practiced the talk aloud imagery method for this scenario, e.g., “chin up, shoulders down, pressed back, I’m dominating.” Hardy et al. (27) found that self-talk during mental imagery is most effective in creating rhythm performance when short phrases or keywords are used to describe actions and thoughts. Therefore, Nina was taught to be more economical in her instructions throughout the exercise by reducing her expressed word count during her talk aloud imagery (i.e., “I will set legs, then pull them in, and then I’ll go for it” to “set, pull, go!”).

Nina, the provider, and her father also discussed her appreciation exchange assignment, where she reported giving appreciation to multiple significant others (e.g., friends, coaches, family). She expressed that this exercise improved her mindset to be “more positively focused” and increased her gratitude for the “small things” in her life.

### 4.4. Meeting 4 (Nina and father)

Nina chose her cast handstand on bars as the performance scenario she desired to optimize for the meeting. As in previous meetings, the provider had Nina utilize the Performance Timeline handout to identify factors that influenced her performance the most and she rehearsed optimal thoughts and behaviors out loud for each of the different performance moments leading up to her cast handstand on bars. Di Corrado et al. (15) found that integrating multiple senses in performance imagery helps improve sports performance. Therefore, the provider targeted Nina's mindfulness and objective thinking skills by having her describe what she was sensing during her Performance Imagery. For example, Nina reported what she was seeing (e.g., father in the crowd), what she smelled and felt (e.g., chalk on her hands), what she heard (e.g., encouragement from teammates).

### 4.5. Meeting 5 (Nina and father)

In this meeting the motivational sandwich was added into Nina's Performance Mapping intervention. The motivational sandwich intervention has been shown to optimize the performance and preparation of athletes (28). She was shown a list of standardized motivational categories with examples listed in each category, and she was asked to rank each category in its ability to motivate herself (see Appendix C). She used this list to construct her motivational sandwich (i.e., state a motivational word, state an instruction, state another motivational word). Nina's performance scenario for this meeting was her cast handstand performance, and her motivational sandwich was, “I’m improving each day; Focus getting shoulders over bar; I’m determined.” She and her father also discussed which motivational statements he could implement during her sport performances and performances outside of sports.

### 4.6. Meeting 6 (Nina and father)

Nina's performance target for this meeting was her back handspring training. During her Performance Mapping imagery, the provider instructed her to imagine aloud stimuli she sensed as leading to feelings of power and motivation. For instance, the provider reported, “You hear your parents in the crowd, and you feel strong and supported,” and she repeated these statements in first person and present tense (e.g., “I hear my parents in the crowd, and I feel strong and supported.”). This approach of repeating optimal statements is aligned with paired-associate learning, which has been shown to improve athletes' skill development (29).

### 4.7. Meetings 7 & 8 (Nina and father)

Nina selected her bar routine and Yurchenko, respectively, for Performance Mapping trials in the 7th and 8th meetings. She implemented her rehearsal with little feedback from the provider and was assigned to practice Performance Mapping prior to bed after each trial. The following dialogue demonstrates Performance Mapping:

*Nina: “I feel the textured floor and begin diaphragmatic breathing… Gonna rock it… Going powerful, wait, turn over, roundoff… Excited to do this skill…”*

*Provider: “You hear your teammates applause, and it makes you feel unstoppable. You smell the rubber as you’re climbing out of the pit, and it reminds you that you're having a blast.”*

*Nina: “I hear my teammates giving me applause, and I feel happy, proud, comfortable and unstoppable.”*

*Provider: “Yes you do!” You're killing it!”*

*Nina: “I smell the blocks and the smell energizes me. I’m having a blast, and I’m going to kill it! ….. After practice my parents tell me I was dominant, and they lead me to feel strong and secure.”*

*Provider: “You are killing it. Go back to this experience and talk about your parents telling you that you’re gonna dominate and how they lead you, but in present tense.”*

The 8th meeting ended with the provider asking Nina to report cognitive and behavioral skills she optimized in the program. Both Nina and her father reiterated that she had improved her ability to think and speak objectively and positively about her training and performance. Nina emphasized that she enjoyed watching how professional gymnasts used diaphragmatic breathing and confident postures like the ones she practiced in her Performance Mapping images. The provider descriptively praised Nina for her engagement and effort in each meeting and her father's willingness to support her throughout the process. Lastly, Nina and her father mentioned that their bond had improved tremendously since the start of the program. She was instructed to continue Performance Mapping prior to bed, and whenever she needed a boost in motivation.

### 4.8. Intervention integrity

To ensure implementation integrity, several strategies were employed, including documentation of techniques used during each meeting, Nina's ratings of engagement and progress towards personal and programmatic goals; ongoing review of meeting audio-tapes by a licensed psychologist and corrective feedback; structured agendas and detailed protocol checklists to guide intervention and measure protocol adherence.

Intervention integrity scores from independent raters were calculated in a two-step process:

1. The overall percentages of intervention protocol steps completed as per the provider's self-report was computed, thus serving as validity estimates for protocol adherence.
2. Ten percent of the session audiotapes rated by the provider for intervention completion were randomly selected and reviewed by independent raters. Inter-rater agreement was computed by adding the number of steps agreed upon by the provider and independent rater and dividing this result by the number of steps agreed upon and disagreed upon by the provider and independent rater and multiplied by 100. Seventy percent protocol adherence and inter-rater agreement is considered satisfactory.

Overall protocol adherence was 98% and inter-rater agreement was 98%; thus, protocols were reliably implemented with high integrity.

#### 4.8.1. Consumer satisfaction and engagement ratings

Following intervention completion, Nina reported high satisfaction with the intervention components, as indicated by the Athlete Helpfulness Rating Scale with an average score of 6.92 (*SD* = .21). The provider rated Nina's engagement with each intervention component, based on attendance/promptness, participation, conduct, and home assignment completion, as being 98% optimal. She also reported high satisfaction with services received, as indicated by the post-intervention CSQ-8 total score of 32. Nina attended 100% of the scheduled meetings.

### 4.9. Complicating factors

There is a need to develop bi-directional mental health assessment measures capable of assessing progress beyond the absence of pathology (7). This is a concern when assessing the impact of optimization programs, such as the current evaluation. Indeed, Nina demonstrated significant decreases in various problem behaviors across several outcome measures in this study. However, it was not possible to assess the extent to which these improvements were optimal (i.e., beyond the absence of psychopathology). Development and utilization of optimization scales are warranted.

### 4.10. Access and barriers to care

There were minor adjustments that had to be made to facilitate video-conferencing (e.g., lower camera to see Nina's stomach while she practiced diaphragmatic breathing, disrupted. connection). However, the benefits of video-conferencing included improvements in access to care due reducing travel time, limiting potential for COVID-19 contraction, and rapid electronic transmission of therapeutic worksheets/handouts.

## 5. Follow-up and outcomes (post-intervention and 3-month follow-up)

The RCI (30) was used to consider the significance of SCL-90-R and SIC assessment score improvements from pre-intervention to post-intervention. The RCI helps determine if the clinical change is significant beyond the standard error of measurement. RCI scores greater than 1.96 reflect changes in scores that are meaningful. Reliable Change Index scores for the SCL-90-R and SIC are listed in Table 1.

**Figure 2:**
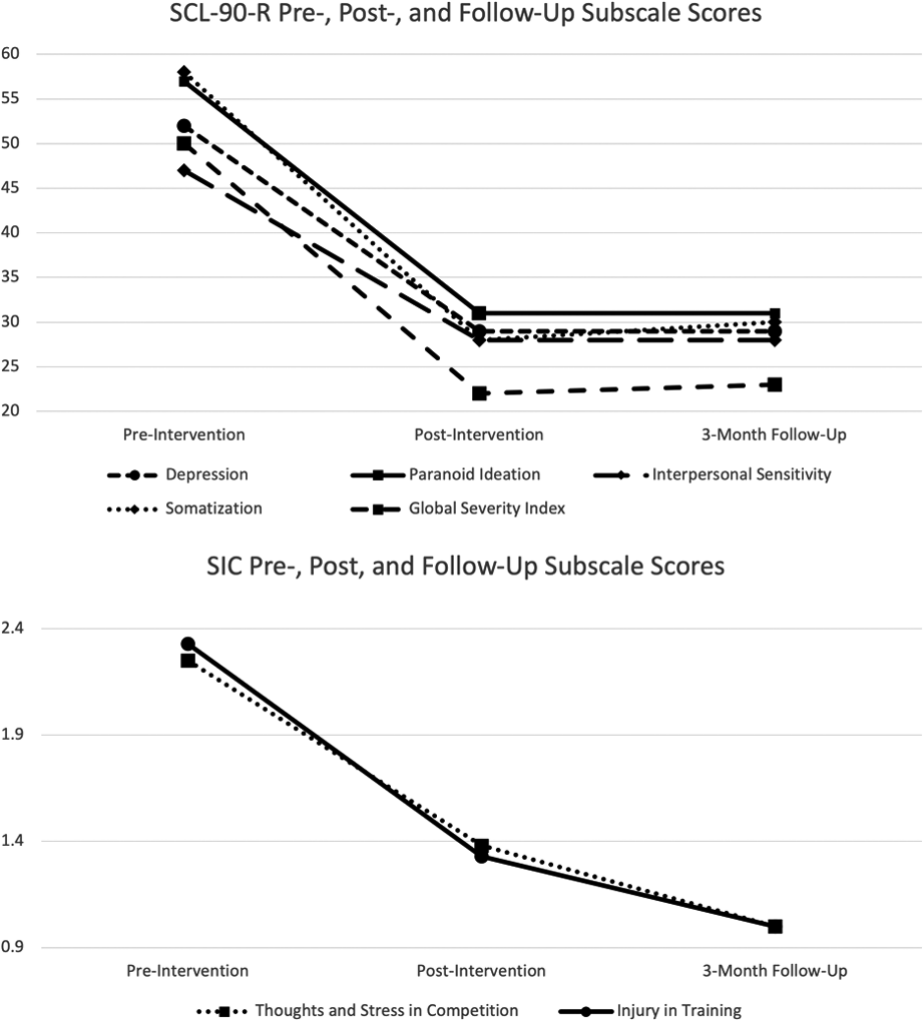
Pre-, post- and follow-up assessments of SCL-90-R and SIC subscales.

Nina evidenced reductions in her SCL-90-R Global Severity Index, depression, paranoid ideation, interpersonal sensitivity, and somatization subscales scales from pre- to post-intervention and from pre-intervention to 3-month follow-up (see Figure 2). More specifically, Nina initially indicated being bothered “quite a bit” by the items “Worrying too much about things” and “Soreness of your muscles.” However, she reported not being bothered by these items at the post-assessment and 3-month follow-up.

Regarding factors interfering with her sports performance, Nina also demonstrated significant improvements on the Thoughts and Stress in Competition and Injury in training subscale on the SIC (see Figure 2). She initially rated being “overly concerned or worried about what others think about her performance,” “often” during training, and “always” during competitions. However, at the post-intervention and 3-month follow-up assessment, she indicated “never” being “overly concerned or worried about how others think about her performance” in training and competition, which contributed to reductions in her Thoughts and Stress in Competition subscale scores. Consistent with her SIC score improvements, Nina also verbally reported improved confidence in practicing and performing new skills in gymnastics. However, a limitation of this study is a lack of measures specific to her physical capacity (e.g., muscle growth) related to her athletic performance.

Eyeballing procedures (31) were used to estimate the magnitude of effect for her SARI scores from pre- to post-test and from pre-test to 3-month follow-up. Post- and follow-up outcome measures demonstrated improvements from baseline for her SARI scores, indicating improved relationships with others (See Table 1). Anecdotally, Nina emphasized at the end of treatment and follow-up that her confidence had been positively affected, and her mindset and gymnastics approach had improved since the start of the program.

## 6. Discussion

This study preliminarily supports Performance Mapping as a potentially efficacious intervention for factors interfering with sport performance and mental health. Moreover, results of the current study (involving an adolescent who did not evidence a mental health disorder) are consistent with previous evaluations of TOPPS that have focused on athletes with, and without mental health disorders. However, in the current study, TOPPS was more focused on the implementation of sport-specific scenarios (i.e., scenarios outside of sport were not explicitly reviewed during performance meetings). This finding supports the assumption that performance optimization programs that exclusively focus on cognitive and behavioral skill acquisition that are directly relevant to sport scenarios may be inherently helpful in the improvement of mental health while reducing stigma that is often associated with the pursuit of traditional psychologically-based mental health treatment programs (32). This assumption will need to be definitively determined in controlled trials, but does point to the importance of respecting qualifications and training of various fields of professional practice when concurrently targeting sport performance and mental health (33). The implications of such findings support the work of qualified sport performance professionals (e.g., AASP Certified Mental Performance Consultants) in the improvement of athletes’ mental health, and further support their collaboration with licensed clinical psychologists (34). For instance, Schinke et al. (35) encouraged licensed clinical psychologists to provide mental health assessments and guide certified mental performance professionals if mental health concerns arise. Moreover, sport performance professionals can provide workshops or training to licensed generalist psychologists to help them better understand the unique benefits of sport and stressors that athletes experience (36). This collaborative model provides economic opportunities for sport performance professionals and licensed clinical psychologists to work interactively to service more athletes with their mental health (37).

A limitation of this study concerns the absence of physical assessments that may have interacted with changes in performance, both in sport and life outside of sport. Future studies, of TOPPS and other performance-focused interventions should include such measures to yield more sophisticated outcome conclusions.

## Data Availability

The original contributions presented in the study are included in the article/Supplementary Material, further inquiries can be directed to the corresponding author.

## Appendix A

**Table:**

**Days Before Situations/Events/Competitions**	**Minutes Before** **Situations/Events/Competitions**	**Seconds Before** **Situations/Events/Competitions**	**During Situations/Events/Competitions**	**Seconds After** **Situations/Events/Competitions**	**Minutes After** **Situations/Events/Competitions**	**Days After** Situations**/Events/Competitions**
**Performance Factors**						
□ Body/physical □ Eating/drinking □ Routines □ Interactions w/others □ Thoughts □ Interpretation of Perceptions (hear, see, smell, taste, feel) □ Emotions □ Energy/motivation □ Training/strategy □ Skills □ Other**:**	□ Body/physical □ Eating/drinking □ Routines □ Interactions w/others □ Thoughts □ Interpretation of Perceptions (hear, see, smell, taste, feel) □ Emotions □ Energy/motivation □ Training/strategy □ Skills □ Other**:**	□ Body/physical □ Eating/drinking □ Routines □ Interactions w/others □ Thoughts □ Interpretation of Perceptions (hear, see, smell, taste, feel) □ Emotions □ Energy/motivation □ Training/strategy □ Skills □ Other**:**	□ Body/physical □ Eating/drinking □ Routines □ Interactions w/others □ Thoughts □ Interpretation of Perceptions (hear, see, smell, taste, feel) □ Emotions □ Energy/motivation □ Training/strategy □ Skills □ Other**:**	□ Body/physical □ Eating/drinking □ Routines □ Interactions w/others □ Thoughts □ Interpretation of Perceptions (hear, see, smell, taste, feel) □ Emotions □ Energy/motivation □ Training/strategy □ Skills □ Other**:**	□ Body/physical □ Eating/drinking □ Routines □ Interactions w/others □ Thoughts □ Interpretation of Perceptions (hear, see, smell, taste, feel) □ Emotions □ Energy/motivation □ Training/strategy □ Skills □ Other**:**	□ Body/physical □ Eating/drinking □ Routines □ Interactions w/others □ Thoughts □ Interpretation of Perceptions (hear, see, smell, taste, feel) □ Emotions □ Energy/motivation □ Training/strategy □ Skills □ Other**:**

See Donohue, B., Chow, G. M., Pitts, M., Loughran, T., Schubert, K. N., Gavrilova, Y., & Allen, D. (2014). Piloting a family-supported approach to concurrently optimize mental health and sport performance in athletes. *Clinical Case Studies,* 1-19.

©Copy only with express written permission from Dr. Brad Donohue (bradley.donohue@gmail.com).

## Appendix B

**Performance Mapping**

A. Solicit performance scenario/situation that the ATHL would like to be optimized
B. Solicit what time periods most impacts the optimization of selected performance scenario/situation (use performance timeline worksheet).
C. Solicit which factors impact the optimization of selected performance scenario/situation
D. Solicit what would be optimal for each factor at the selected moment in time
E. Determine specific statements that are optimally motivating for top 3 ranked motivational categories for self and what significant others (SOs) would say that is motivating (use motivational statements questionnaire)
F. Instruct ATHL to objectively describe optimal performance for the respective time period in terms of what ATHL is doing and thinking from start to finish
  1. Interrupt and have ATHL repeat to connect senses, thoughts, actions, and motivational statements for self and SOs in real time
    i. Prompt objective senses resulting in power/passion/positive experiences
      a. Hear, Smell, Taste, Sight, and Touch
    ii. Replace non-optimal thoughts or actions with optimal thoughts and actions immediately
    iii. Descriptively praise optimal thoughts or actions
    iv. Query actions, thoughts, and senses not reviewed to improve accuracy
G. Continue to rehearse desired performance moments until optimal

## Appendix C

©Copy only with express written permission from Dr. Brad Donohue (bradley.donohue@gmail.com)
